# Sodium Butyrate Enhances Curcuminoids Permeability through the Blood-Brain Barrier, Restores Wnt/β-Catenin Pathway Antagonists Gene Expression and Reduces the Viability of Glioblastoma Cells

**DOI:** 10.3390/ijms222011285

**Published:** 2021-10-19

**Authors:** Aleksandra Majchrzak-Celińska, Robert Kleszcz, Anna Stasiłowicz-Krzemień, Judyta Cielecka-Piontek

**Affiliations:** 1 Department of Pharmaceutical Biochemistry, Poznan University of Medical Sciences, 4 Święcicki Str., 60-781 Poznań, Poland; kleszcz@ump.edu.pl; 2 Department of Pharmacognosy, Poznan University of Medical Sciences, 4 Święcicki Str., 60-781 Poznań, Poland; astasilowicz@ump.edu.pl (A.S.-K.); jpiontek@ump.edu.pl (J.C.-P.)

**Keywords:** curcuminoids, sodium butyrate, glioblastoma, cell cycle, apoptosis, reactive oxygen species, DNA methylation, permeability

## Abstract

Glioblastoma (GBM) is an extremely aggressive brain tumor awaiting novel, efficient, and minimally toxic treatment. Curcuminoids (CCM), polyphenols from *Curcuma longa*, and sodium butyrate (NaBu), a histone deacetylase inhibitor naturally occurring in the human body, await elucidation as potential anti-GBM agents. Thus, the aim of this study was to analyze CCM and NaBu both separately and as a combination treatment using three GBM cell lines. MTT was used for cytotoxicity evaluation, and the combination index was calculated for synergism prediction. Cell cycle, apoptosis, and reactive oxygen species (ROS) generation were analyzed using flow cytometry. DNA methylation was verified by MS-HRM and mRNA expression by qPCR. The permeability through the blood-brain barrier (BBB) and through the nasal cavity was evaluated using PAMPA model. The results of this study indicate that CCM and NaBu synergistically reduce the viability of GBM cells inducing apoptosis and cell cycle arrest. These effects are mediated via ROS generation and changes in gene expression, including upregulation of Wnt/β-catenin pathway antagonists, *SFRP1*, and *RUNX3*, and downregulation of *UHRF1*, the key epigenetic regulator. Moreover, NaBu ameliorated CCM permeability through the BBB and the nasal cavity. We conclude that CCM and NaBu are promising agents with anti-GBM properties.

## 1. Introduction

More than 24,500 new cases of the brain & other nervous system tumors and more than 18,500 deaths are estimated to occur in 2021 in the United States [1]. Brain and other nervous system tumors are the leading cause of cancer death among men aged <40 years and women aged <20 years worldwide [1]. Among various brain tumor types, glioblastoma (GBM) is the most common, with an incidence of 3.7 per 100,000 persons per year [2]. It is also the most aggressive primary brain tumor in adults, with an overall 2-year survival of 18%, 3-year survival of 11%, and 5-year survival of only 4% [2]. Numerous factors implement the high aggressiveness of GBM cells, with the hyperactivated Wnt/β-catenin pathway being one of the most important. Thus, novel approaches to the treatment and control of GBM are urgently needed. A growing number of studies indicates that natural bioactive molecules may serve well in this purpose.

Numerous epidemiological studies demonstrated the relationship between diet and cancer and the potential of dietary components, particularly polyphenols as promising anticancer and chemopreventive agents [3,4]. Curcumin, also known as diferuloylmethane (1,7-bis (4-hydroxy-3-methoxyphenyl)-1,6-hepadiene-3,5-dione), is a natural polyphenol extracted from the rhizome of *Curcuma longa*. It was used for centuries in traditional Indian Ayurvedic and Chinese medicine for treating respiratory and liver disorders, digestive disorders, infections, allergies, and rheumatisms [5]. Curcumin has low inherent toxicity and various properties with significant impact and potential medical applications [6]. It was reported to modulate the activity of kinases and transcription factors as well as to regulate the expression of genes involved in cell survival and malignant transformation [5]. Numerous studies show that it regulates multiple cellular signaling pathways, including Wnt/β-catenin, PI3K/Akt, JAK/STAT, MAPK, p53 and NF-ĸB signaling pathways [7]. Curcumin can also epigenetically regulate gene expression by reversing DNA methylation, altering histone modifications, and targeting several miRNAs [8]. Curcumin can cross the blood-brain barrier, thus yielding therapeutic benefits within the CNS [9]. However, its impact on GBM remains to be elucidated before curcumin can enter the clinical trial phase as a potential anti-GBM substance.

Sodium butyrate (NaBu), a salt of short chain fatty acid, is one of the main products of the anaerobic fermentation of indigestible polysaccharides such as dietary fiber and resistant starch produced by the microbiota in the large intestine [10]. It is produced in the lower gastrointestinal tract and was proposed to confer a number of health benefits, including a reduced risk of colorectal cancer [11,12]. Moreover, through the bidirectional communication between the gastrointestinal tract and the central nervous system (CNS), NaBu exerts its neuroactive properties [13]. Animal studies showed that NaBu and other short chain fatty acids exert widespread influence on key neurological and behavioral processes and may be involved in critical phases of neurodevelopmental and neurodegenerative disorders [13]. It has neuroprotective activity and was shown to be effective against several neuropsychiatric disorders [14,15]. Moreover, it was reported to exert antitumor effects in many tumors including not only colorectal cancer [12], but also breast cancer [16], and GBM [17,18]. The reports evaluating NaBu in the context of GBM cells are however scarce and the issue of whether NaBu can be considered as anti-GBM agent requires further investigation. The bidirectional axis between the intestine and the brain was recently highlighted as an important determinant of glioma biology [18], making NaBu an interesting agent for investigation. Moreover, according to our best knowledge, up to date, no study shows the effects of the combined treatment with curcumin, or more generally, curcuminoids (CCM) and NaBu on human GBM cells.

Thus, the aim of this study was to analyze the impact of CCM and NaBu on GBM cell viability and to check whether these compounds act synergistically. Other goals were to analyze the role of CCM and NaBu in inducing cell cycle arrest and apoptosis in GBM cells and to verify if the ability to generate reactive oxygen species (ROS) plays a role in these phenomena. The analysis of DNA methylation and expression changes in genes essential in gliomagenesis (Wnt pathway antagonists, *SFRP1*, *RUNX3* and master cell cycle regulator, *RASSF1A*), and the impact on the expression of the crucial epigenetic regulator *UHRF1* were evaluated to further decipher the molecular mechanisms exerted by CCM and NaBu in GBM cells. Finally, to verify if the two compounds can successfully be implemented as a combination treatment targeting CNS, the permeability of CCM and NaBu through the blood-brain barrier (BBB) and the nasal cavity was analyzed.

## 2. Results

### 2.1. Chemical Composition of CCM

**Figure 1:**
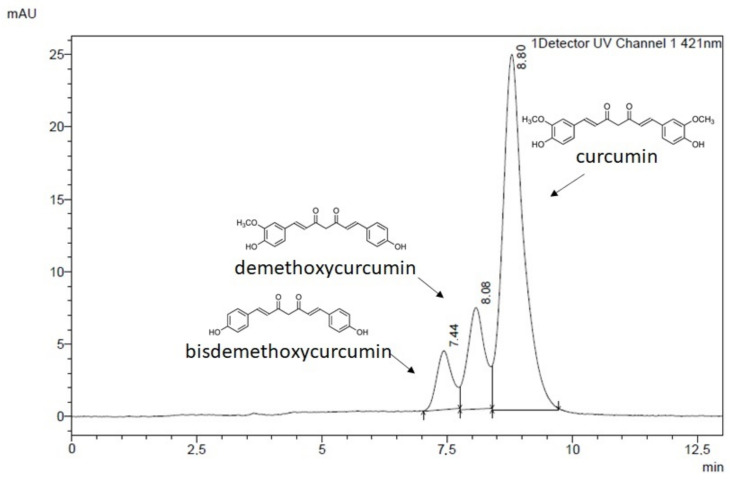
Chromatogram of curcuminoids from Curcuma longa sample showing abundance of curcumin and presence of demethoxy- and bisdemethoxycurcumin.

Typical extracts of *Curcuma longa* L. contain curcumin, demethoxycurcumin, and bisdemethyoxycurcumin, of which curcumin is the most common. Our HPLC analysis revealed that the chemical composition of the analyzed product, ‘Curcumin from *Curcuma longa* (Turmeric)’ (Sigma-Aldrich, St. Louis, MI, USA) contains all three structures (Figure 1).

The most abundant component was curcumin (57.56%), while demethoxycurcumin, and bisdemethoxycurcumin constituted 24.93% and 17.51% of the analyzed sample, respectively. As presented in the Figure 1, the recorded retention times were: 7.440 min for bisdemethoxycurcumin, 8.080 min for demethoxycurcumin, and 8.795 min for curcumin. In this manuscript, the abbreviation CCM is used to describe all three compounds (curcumin, demethoxycurcumin, and bisdemethoxycurcumin) present in the curcumin from *Curcuma longa* chemical substance under investigation.

### 2.2. CCM and NaBu Are Cytotoxic to GBM Cells in a Dose-Depenedent and Cell Line-Dependent Manner

**Figure 2:**
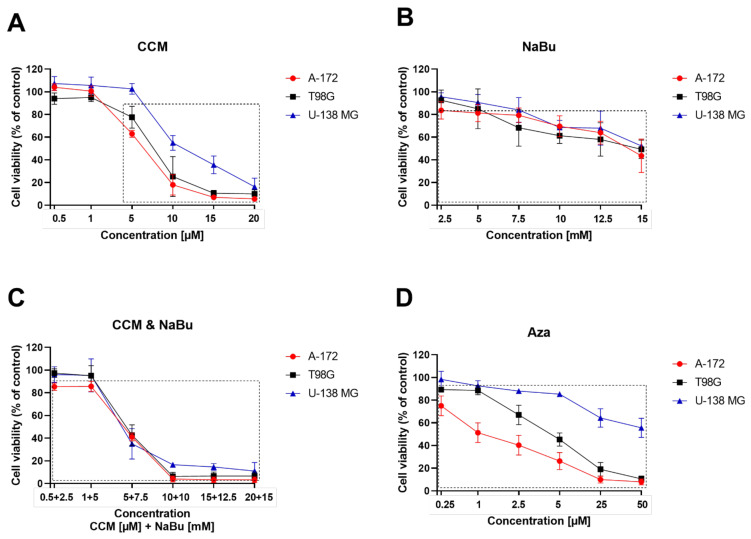
Cytotoxicity evaluation of CCM (Panel **A**), NaBu (Panel **B**), and their combination treatment (Panel **C**), as well as Aza (Panel **D**) based on 48 h MTT test on GBM cell lines, A-172 (red), T98G (black), and U-138 MG (blue). Vehicle-treated cells (control) were assigned as 100% cell viability. Statistically significant difference in cell viability between cells exposed to analyzed compound/combination of compounds as compared to that of control cells is indicated with rectangle. Values are shown as mean ± SEM calculated from three independent experiments.

The cytotoxic effect of CCM, NaBu, and their combination treatment, as well as 5-azacitidine (Aza), was studied as a dose-response MTT experiment conducted for 48 h. As presented in the Figure 2, the tested concentration ranges were: 0.5–20 µM for CCM, 2.5–15 mM for NaBu, and the appropriate combinations of both. Aza, a DNMT-inhibitor used as a reference substance in MS-HRM (methylation-sensitive high-resolution melting) analysis, was tested in a concentration range of 0.25–50 µM.

The obtained results indicate that in the case of CCM treatment, the most sensitive cell line was A-172, and the least sensitive was U-138 MG. In this regard, 5 µM CCM significantly reduced the number of living cells of A-172 cell line (*p* = 0.0003), and T98G (*p* = 0.0430), having no impact on U-138 MG cell line. Contrarily, the highest tested concentration, e.g., 20 µM CCM, was cytotoxic to all the cell lines.

In respect to NaBu, the tested range of concentrations gradually decreased the viability of GBM cells. Still, even the highest tested concentration, e.g., 15 mM was not able to kill all cancer cells—the percentage of living cells was 43.7% for A-172 cell line, 49.3% for T98G cell line, and 52.3% for U-138 MG cell line.

However, the simultaneous treatment with both CCM and NaBu (the combination treatment) was more cytotoxic than single treatments. In this regard, 5 µM CCM and 7.5 mM NaBu reduced the percentage of living cells to ~40% for all the cell lines. In comparison, the subsequent tested combination (10 µM CCM and 10 mM NaBu) almost completely killed the cells, leaving only 4.0%, 6.3%, and 16.7% of living cells of A-172, T98G, and U-138 MG cell line, respectively.

Aza treatment showed variable cytotoxic effects with different cell lines. Again, A-172 cell line was the most sensitive to the treatment, while U-138 MG cell line, the least. In this regard, 50 µM of Aza diminished the percentage of living cells to ~ 8% in A-172 and T98G cells, whereas 55.6% of U-138 MG cell line remained alive in these conditions.

Based on the MTT results of each particular cell line, the concentrations of compounds/combination treatment allowing the survival of more than 70% of cells were chosen for further analysis. In this regard, the concentration of 1 µM CCM was analyzed when A-172 cells were evaluated, while 5 µM CCM was analyzed for T98G and U-138 MG cell lines. NaBu was investigated in a concentration of 7.5 mM in A-172 and U-138 MG cells, while 5 mM was used in T98G cells. The combination treatment consisted of 1 µM CCM and 5 mM NaBu, irrespective of the cell line. Aza was evaluated in 0.25 µM, 1 µM, and 5 µM, for A-172, T98G, and U-138 MG cells, respectively.

### 2.3. The Combination Treatment of CCM and NaBu Synergistically Decreases the Viability of GBM Cells

**Figure 3:**
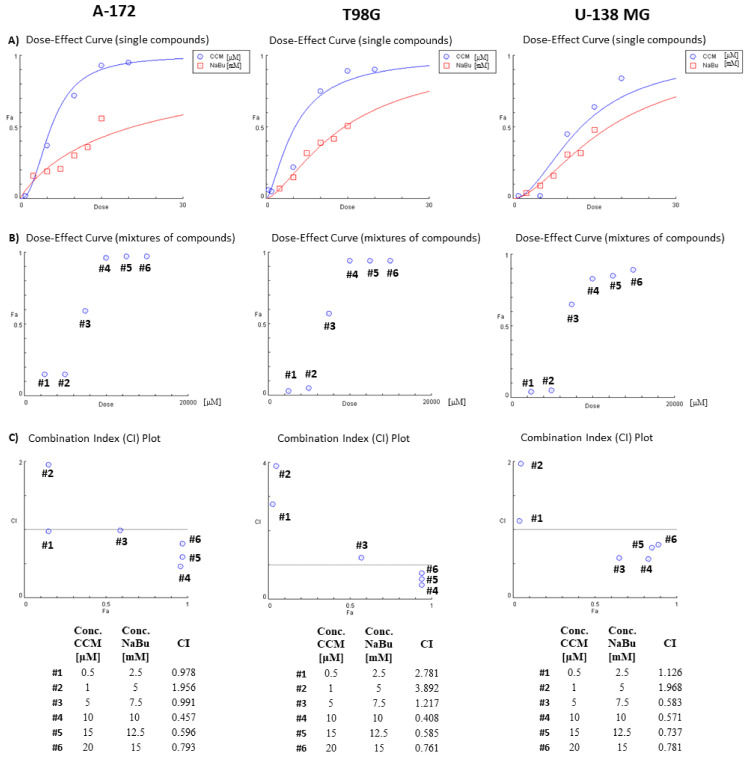
Results of analysis of combinatorial effects of CCM and NaBu and their combination treatment on GBM cell viability based on MTT assay. A-172, T98G, and U-138 MG cells were treated with increasing concentrations of individual compounds and their combinations for 48 h. Control cells were treated with vehicle. Mean values from three independent experiments were used in calculations using CompuSyn software. Dose-effect curves for individual compounds (panel **A**) or combination treatment (panel **B**) were generated. (Panel **C**) presents evaluation of the combination index (CI). Synergistic action of chemicals in combinations was identified when CI ˂ 1 (the area below the grey line in Panel **C** graphs). Synergistic interactions in terms of the reduction of cell viability were obtained for concentrations higher than 1 µM CCM and 5 mM NaBu. Fa—Fraction affected: viability reduction from lack of effect (0) to maximal effect (1; viability = 0%).

Next, we wanted to evaluate if CCM and NaBu act synergistically regarding cytotoxic effects on GBM cells. As presented in the Figure 3, the analysis of the combinatorial effect of the compounds on cell viability revealed that CCM and NaBu synergistically reduce the viability of GBM cells when the concentrations used are higher than 1 µM CCM and 5 mM NaBu. The synergistic combinations included, in case of A-172 and U-138 MG cell lines 5 µM CCM and 7.5 mM NaBu, and in respect to all three cell lines, the combinations of 10 µM CCM and 10 mM NaBu, 15 µM CCM and 12.5 mM NaBu, as well as 20 µM CCM and 15 mM NaBu (Figure 3C).

### 2.4. CCM, NaBu, and Their Combination Treatment Induce Apoptotic Cell Death of GBM Cells

**Figure 4:**
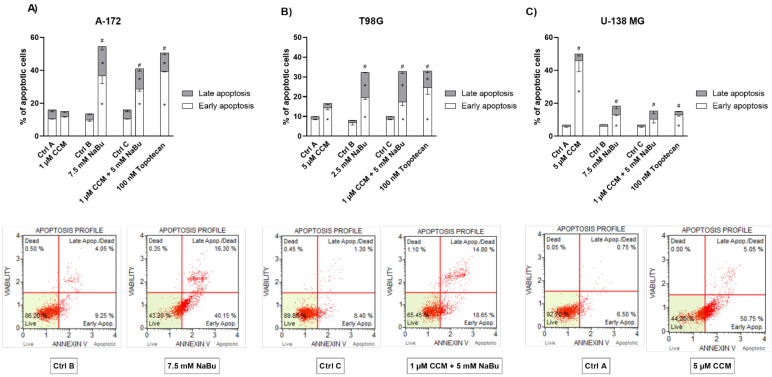
Effect of CCM and NaBu and their combinations on apoptosis detected in A-172 (**A**), T98G (**B**), and U-138 MG (**C**) cell lines after 48 h of treatment. Cells treated with vehicle (DMSO, PBS or both) are designated as Ctrl A, Ctrl B, and Ctrl C, respectively. In addition to each figure showing percentage of cells in early and late apoptosis phase, lower figures show histogram of compound/combination treatment with most significant proapoptotic effect compared to that of negative control (i.e., Ctrl B and 7.5 mM NaBu refer to A-172 cell line, Ctrl C and 1 µM CCM and 5 mM NaBu refer to T98G cell line, and Ctrl A and 5 µM CCM refer to U-138 MG cell line). Values are shown as mean ± SEM calculated from three independent experiments. (*) indicates statistically significant differences from control group for early/late apoptosis, (#) above bar indicates statistically significant differences from control group for total apoptotic cells, *p* < 0.05.

The results of apoptosis analysis revealed that CCM and NaBu used as single treatments can induce apoptosis in GBM cells, but their combination treatment is also effective. However, different cell lines reacted slightly differently to the treatments. Concerning A-172 cell line, the most potent proapoptotic effect was obtained after the treatment with 7.5 mM NaBu (Figure 4A). In fact, the impact of NaBu was as strong as the one obtained using the anticancer drug—topotecan, used as a positive control in this assay. In this cell line, CCM did not increase the percentage of apoptotic cells. Nevertheless, the combination treatment of CCM and NaBu were still acting proapoptotically.

The results for T98G cell line were generally similar to those obtained for A-171 cells, with the exception that here the treatment with CCM increased the number of cells in the early apoptotic phase (Figure 4B). However, when both early and late apoptotic cells were counted together, their number was not significantly higher as compared to that of the control. Here, again NaBu and its combination with CCM were the most effective, and their effect was comparable to that of 100 nM topotecan.

Contrarily, U-138 MG cell line was the most susceptible to the treatment with CCM (Figure 4C). In this regard, the effect of 5 µM CCM was more than twice as strong as 100 nM topotecan. NaBu and its combination treatment with CCM also significantly induced apoptosis, but their impact was less pronounced.

### 2.5. CCM and NaBu Alter the Distribution of the Cell Cycle Phases

**Figure 5:**
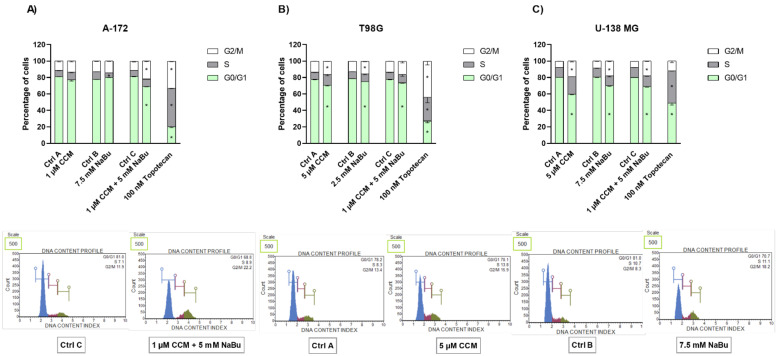
Impact of CCM and NaBu and their combination on cell cycle distribution in A-172 (Panel **A**), T98G (Panel **B**), and U-138 MG cells (Panel **C**). Percentage of cells in G1/G0, S, and G2/M phase was analyzed by flow cytometry after staining with propidium iodide, and RNase A. Topotecan was used as a positive control in this assay. Cells treated with vehicle (DMSO, PBS, or both) are designated as Ctrl A, Ctrl B, and Ctrl C, respectively. Representative histograms are shown below diagrams (Ctrl C and combination treatment of 1 µM and 5 mM NaBu refer to A-172 cell line, Ctrl A, and 5 µM CCM refer to T98G cell line, and finally Ctrl B and 7.5 mM NaBu refer to U-138 MG cell line). Values are shown as mean ± SEM calculated from three independent experiments. (*) indicates statistically significant differences from control group for a particular phase, *p* < 0.05.

The analysis of the cell cycle distribution revealed that the observed effects are also cell line-dependent. CCM in a concentration of 1 µM did not induce the changes in the distribution of the cell cycle phases in A-172 cell line (Figure 5A). Contrarily, NaBu treatment decreased the number of cells in the S phase, with no significant impact on the other analyzed cell cycle phases. Interestingly, the combination treatment of CCM and NaBu diminished the percentage of cells in G0/G1 cell phases and increased the percentage of cells in G2/M phases.

In contrast, in T98G cell line, both CCM and NaBu effectively induced cell cycle arrest when administered as single compounds (Figure 5B). The effect observed after the treatment with CCM and NaBu was very similar—both compounds decreased the number of cells in the G0/G1 phases, increasing the number of cells in the G2/M phases. A comparable effect was observed when both compounds were added in a combination (here, however, only the decrease of percentage of cells in G0/G1 phase of the cell cycle was statistically significant).

Similar effects were reported for U-138 MG cell line. CCM and NaBu, used as single compounds and as a combination treatment shifted the cells from G0/G1 phase to G2/M phase, halting the cell cycle. The effects resembled those of 100 nM topotecan, but were less pronounced (Figure 5C).

### 2.6. CCM, NaBu, and Their Combination Treatment Induce Oxidative Stress in GBM Cell Lines

**Figure 6:**
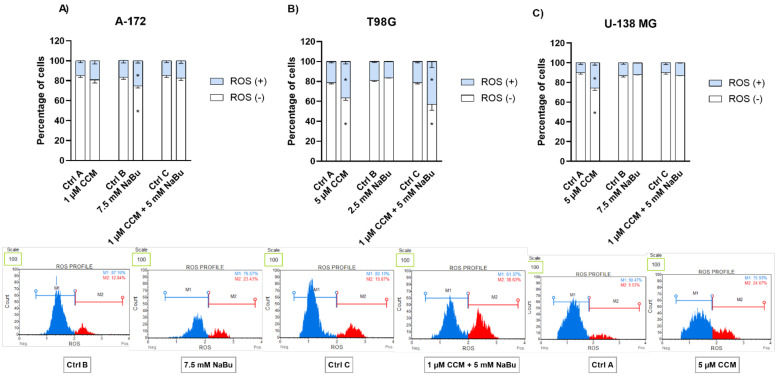
Impact of CCM and NaBu, and their combination on ROS (superoxide) generation in A-172 (**A**), T98G (**B**), and U-138 MG (**C**) cell lines after 48 h of treatment. Percentage of ROS positive (ROS (+)) and negative (ROS (−)) was detected by flow cytometric analysis after staining with dihydroethidium. Cells treated with vehicle (DMSO, PBS or both) are designated as Ctrl A, Ctrl B, and Ctrl C, respectively. Exemplary histograms are presented below diagrams (Ctrl B and 7.5 mM NaBu refer to A-172 cell line, Ctrl C and combination treatment of 1 µM and 5 mM NaBu refer to T98G cell line, and Ctrl A and 5 µM CCM refer to U-138 MG cell line). Values are shown as mean ± SEM calculated from three independent experiments. (*) indicates statistically significant differences from control group, *p* < 0.05.

The flow cytometry analysis of ROS production revealed that CCM and NaBu used as single compounds, as well as a combination treatment, were able to induce oxidative stress in GBM cells. However, the effects were cell line-specific. In this regard, NaBu increased the percentage of ROS (+) cells in A-172 cells (Figure 6A), while CCM generated ROS production in both T98G and U-138 MG cells (Figure 6B and Figure 6C, respectively). Moreover, the combination treatment generated a significant amount of superoxide radicals in T98G cells.

### 2.7. NaBu and the Combination Treatment of CCM and NaBu Significantly Increase the Expression of Wnt Pathway Antagonists and Downregulate the Epigenetic Regulator, UHRF1

**Figure 7:**
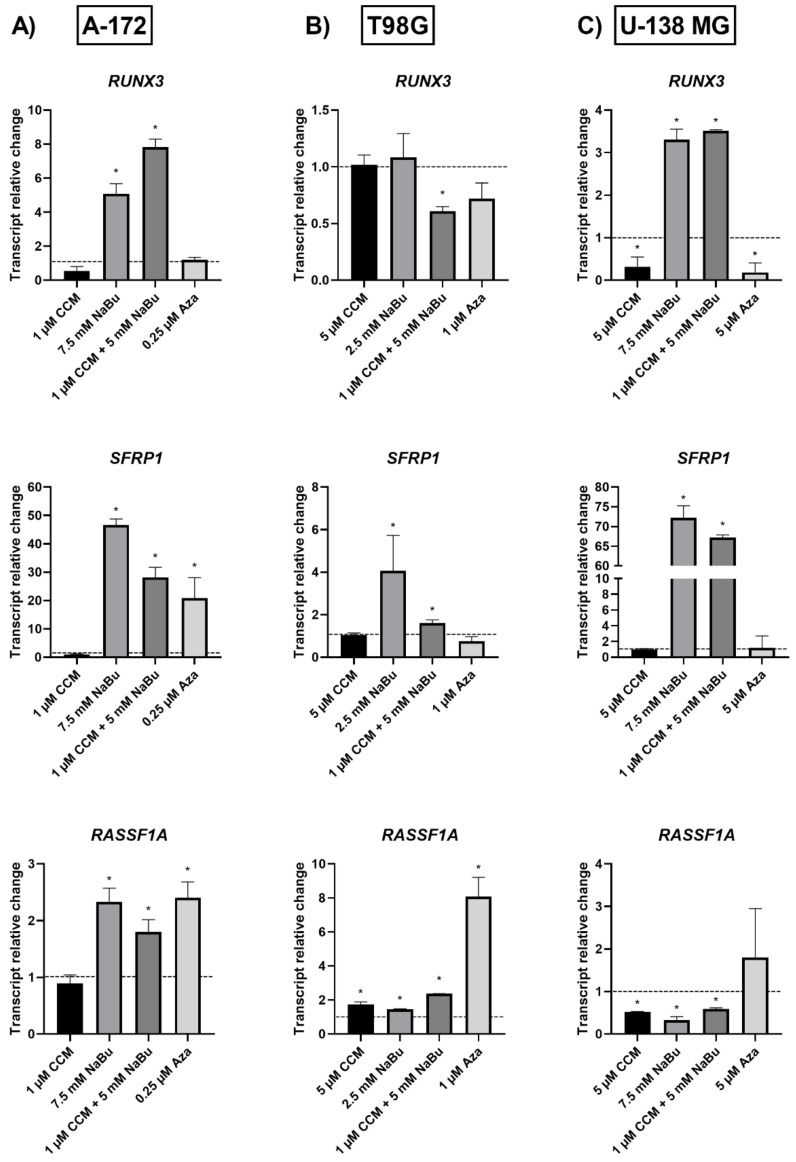
Transcript relative change of Wnt pathway antagonists, *RUNX3*, *SFRP1*, and cell cycle regulator, *RASSF1A* in A-172 (**A**), T98G (**B**), and U-138 MG (**C**) cells after treatment with CCM, NaBu, combination treatment of CCM & NaBu, and Aza. Data are presented as mean values ± SEM from two independent experiments. Control equals 1 and is indicated with a horizontal line. (*) above bar indicates statistically significant differences from control group, *p* < 0.05.

To evaluate if CCM and NaBu influence the expression of Wnt pathway antagonists, we analyzed the mRNA level of *RUNX3* and *SFRP1* (Figure 7). Here, we report that CCM did not alter the expression of both genes, except U-138 MG cell line, where it downregulated *RUNX3*. In contrast to CCM, NaBu significantly upregulated both genes. Around 5-fold increase in the expression of *RUNX3* was observed in A-172 and ~3.3 fold in U-138 MG cell line.

The transcript level of *SFRP1* was even more elevated—its mRNA level increased 46.6-times in line A-172, 4.0-times in T98G, and 72.2-times in U-138 MG cell line after the treatment with NaBu. The combination treatment of CCM and NaBu was also effective in upregulating this Wnt pathway antagonist. It increased the mRNA level of both *RUNX3* and *SFRP1* in all three cell lines analyzed. In comparison, Aza significantly increased *SFRP1* transcript level in A-172 cell line, but in T98G and U-138 MG cell lines its impact was inconsiderable.

Next, we evaluated *RASSF1A* expression (Figure 7). In this context, CCM upregulated its expression in T98G cells, but the opposite effect was observed in U-138 MG cell line. The impact of NaBu and the combination treatment resembled that of CCM. These compounds upregulated *RASSF1A* not only in T98G cells, but also in A-172, and downregulated its expression in U-138 MG cells. Aza increased its mRNA level in both A-172 and T98G cell lines, but a trend towards upregulation was also observed in U-138 MG cells.

**Figure 8:**
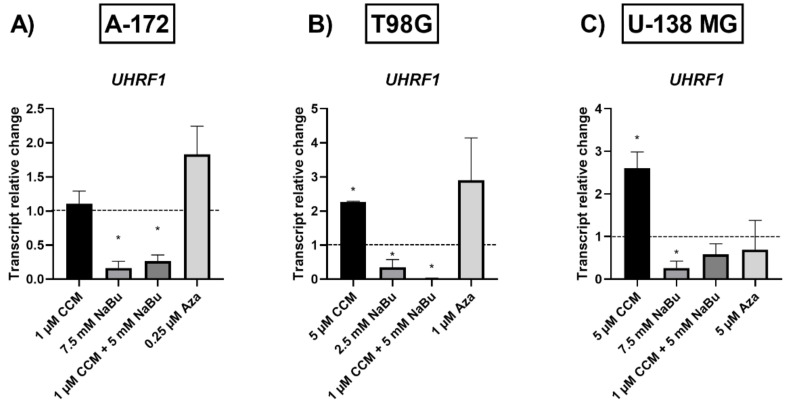
Transcript relative change of epigenetic regulator *UHRF1* in A-172 (**A**), T98G (**B**), and U-138 MG (**C**) cells after treatment with CCM, NaBu, combination treatment of CCM & NaBu, and Aza. Data are presented as mean values ± SEM from two independent experiments. Control equals 1 and is indicated with a horizontal line. (*) above bar indicates statistically significant differences from control group, *p* < 0.05.

Finally, the impact of the analyzed compounds was also evaluated in regard to the UHRF1 expression (Figure 8). CCM increased its mRNA level in T98G and U-138 MG cells, while NaBu and the combination treatment had the opposite effect—a significant decrease of *UHRF1* transcript was observed in all the three cell lines (with the exception for U-138 MG cell line and the combination treatment, where the result was not significant). Aza did not alter *UHRF1* expression, but a trend towards upregulation was observed in A-172 and T98G cell lines.

### 2.8. CCM and NaBu Do Not or Only Mildly Affect DNA Methylation Level of RUNX3, SFRP1, and RASSF1A Promoters

**Figure 9:**
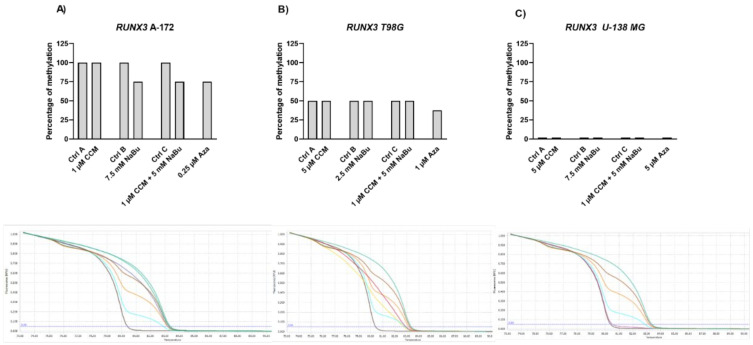
Results of MS-HRM analysis with representative melting curves of *RUNX3* promoter methylation in A-172 (**A**), T98G (**B**), and U-138 MG (**C**) cell lines. Cells treated with vehicle (DMSO, PBS or both) are designated as Ctrl A, Ctrl B, and Ctrl C, respectively. Due to semiquantitative nature of generated results, no SEM bars are shown. Below each bar chart melting curves of control and best demethylating compound/combination is presented, together with subsequent controls: 100%, 75%, 50%, 25%, and 0% methylated controls (upper green, brown, honey-yellow, blue, and dark brown, respectively). For A-172 cell line, melting curve generated after treatment with 1 µM CCM + 5 mM NaBu mix is presented (blue line) regarding Ctrl A (green line, below 100% methylated DNA). For T98G cell line, Aza is presented (fair yellow) regarding Ctrl A (red). In case of U-138 MG cell line, only Ctrl A is presented (pink), reflecting no DNA methylation in analyzed region.

Our MS-HRM analysis revealed that *RUNX3* was methylated in A-172 cells (100% methylation) and unmethylated in U-138 MG cells, whereas in T98G cell line, its methylation level reached 50%, due to the presence of both methylated and unmethylated alleles (Figure 9). The results indicate that 48 h treatment with CCM did not demethylate the above-mentioned promoter. In contrast, NaBu and the combination treatment of CCM and NaBu slightly reduced the methylation level of *RUNX3* in the A-172 cell line (from 100% to 75%). Similar results were obtained after the treatment with Aza—the methylation level of *RUNX3* dropped slightly, so the HRM curve reached the level of 75% in A-172 cell line, and was in the range of 25–50% in T98G cells.

**Figure 10:**
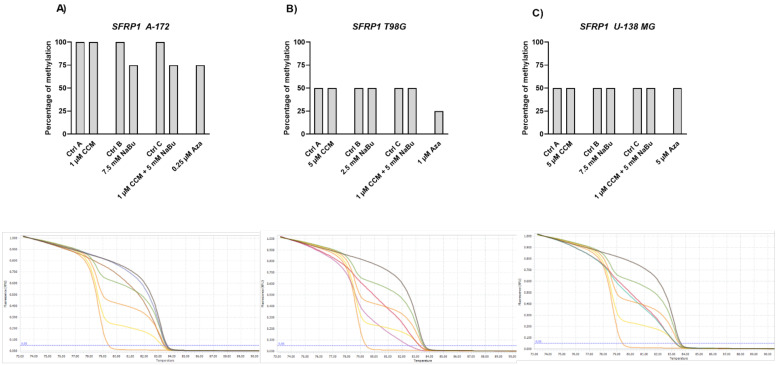
Results of MS-HRM analysis with representative melting curves of *SFRP1* promoter methylation in A-172 (**A**), T98G (**B**), and U-138 MG (**C**) cell lines. Cells treated with vehicle (DMSO, PBS or both) are designated as Ctrl A, Ctrl B, and Ctrl C, respectively. Due to semiquantitative nature of generated results, no SEM bars are shown. Below each bar chart melting curves of control and best demethylating compound/combination are presented, together with subsequent controls: 100%, 75%, 50%, 25%, and 0% methylated controls (dark brown, green, honey yellow—middle line, bright yellow, and honey yellow—lower line, respectively). For A-172 cell line, melting curve generated after treatment with 1 µM CCM + 5 mMNaBu mix is presented (mustard brown line, crossing the 75% green line) regarding Ctrl A (blue). For T98G cell line, Aza is presented (pink) in relation to Ctrl A (red). In case of U-138 MG cell line, 5 µM CCM is shown (green) regarding Ctrl A (red).

A second Wnt pathway antagonist analyzed in this study, *SFRP1*, was found to be fully methylated in A-172 cell line, and hemimethylated in both T98G and U-138 MG cells (Figure 10). Aza hypomethylated *SFPR1* promoter in A-172 and T98G cell lines, having no impact on U-138 MG cells. The observed methylation level decrease was from 100% to 75% in A-172 cell line, and from 50% to ~25% in T98G cell line. Similar effects were reported for NaBu and its combination treatment with CCM in A-172 cells. No changes in DNA methylation level of *SFRP1* were, however, detected in U-138 MG cell line. Also, the treatment with CCM did not alter the methylation level of the analyzed sequence in any of the tested cell lines.

**Figure 11:**
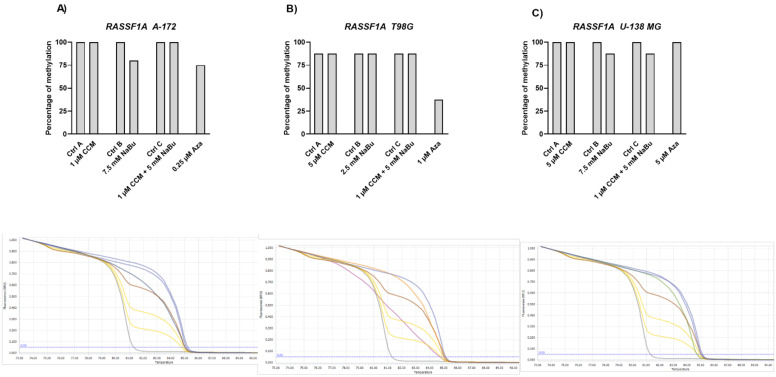
Results of MS-HRM analysis with representative melting curves of *RASSF1A* promoter methylation in A-172 (**A**), T98G (**B**), and U-138 MG (**C**) cell lines. Cells treated with vehicle (DMSO, PBS or both) are designated as Ctrl A, Ctrl B, and Ctrl C, respectively. Due to semiquantitative nature of generated results, no SEM bars are shown. Below each bar chart melting curves of control and best demethylating compound/combination are presented, together with subsequent controls: 100%, 75%, 50%, 25%, and 0% methylated controls (blue, brown, upper yellow, lower yellow, grey, respectively). For A-172 cell line, melting curve generated after treatment with 5-Aza is presented (dark blue, close to brown, 50% methylated control) regarding Ctrl A (blue line, close to 100% methylated control). For T98G cell line, also Aza is presented (pink) regarding Ctrl A (orange). In case of U-138 MG cell line, 7.5 mM NaBu is presented (green) in relation to Ctrl B (blue line, close to 100% methylated control).

Finally, we wanted to analyze if the *RASSF1A* methylation level is altered after the treatment with CCM and NaBu, and their combination treatment (Figure 11). Our results indicate that this tumor suppressor gene was fully methylated (100% methylation) in A-172 and U-138 MG cell lines, whereas in T98G cell line, it reached the range of 75–100% methylation. We found that the *RASSF1A* methylation level was diminished when Aza was used (in A-172 and T98G cells), while U-138 MG cells were resistant to its hypomethylating properties. The greatest (~50%) decrease of DNA methylation level was observed in T98G cell line, after the treatment with 1 µM Aza. Similarly, as in the case of the other analyzed genes, CCM was ineffective in *RASSF1A* demethylation. However, NaBu mildly demethylated the promoter region of *RASSF1A* in A-172 and U-138 MG cells and, in the latter cell line, the combination treatment of CCM and NaBu was also effective.

### 2.9. NaBu Increases the Permeability of CCM through the Blood-Brain Barrier (BBB) and the Nasal Epithelium

**Figure 12:**
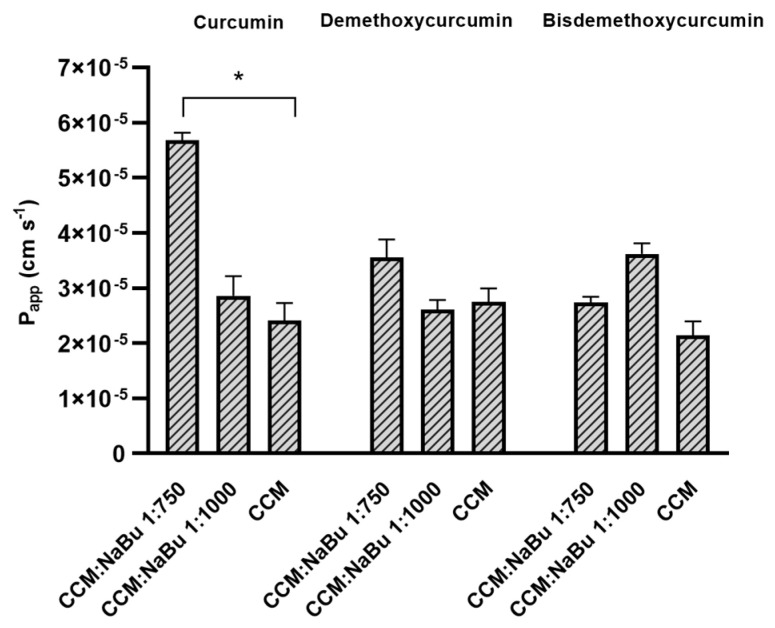
Values of apparent permeability coefficients of curcumin, demethoxycurcumin, and bisdemethoxycurcumin alone and with an addition of NaBu in the molar ratio of 1:750 (20 µM CCM:15 mM NaBu) and 1:1000 (10 µM CCM:10 mM NaBu) determined for permeability through blood-brain barrier. Values are shown as mean ± SEM calculated from three independent experiments. (*) indicates statistically significant differences, *p* < 0.05.

To analyze the permeability of CCM and NaBu through the BBB, the CCM sample was studied alone and with NaBu in molar ratio of CCM:NaBu 1:750; and 1:1000, following the ratios of the synergistic combination treatments (10 µM CCM:10 mM NaBu and 20 µM CCM:15 mM NaBu, respectively). The permeability coefficients (P*_app_*) of curcuminoids: curcumin, demethoxycurcumin, and bisdemethoxycurcumin, through the BBB were within the range of 2.140 × 10^−5^ to 2.751 × 10^−5^ cm s^−1^ and were very similar to one another (Figure 12). Moreover, all of the compounds had P*_app_* values greater than 4.0 × 10^−6^ cm s^−1^, proving their good permeability through the BBB [19]. Importantly, the use of CCM:NaBu combination in the ratio 1:750 increased the permeability of curcuminoids through the BBB. Regarding CCM, an over 2.3-fold increase in permeability was observed (P*_app_* 5.684 × 10^−5^ ± 1.333 × 10^−6^ cm s^−1^). Addition of NaBu in a ratio of CCM:NaBu 1:750 also resulted in an increase in demethoxycurcumin permeability (P*_app_* 3.557 × 10^−5^ ± 3.266 × 10^−6^ cm s^−1^); however, the result was not statistically significant. A mild increase in the permeability of bisdemethoxycurcumin through the BBB was recorded after using the combination of CCM:NaBu in the ratio 1:1000 (P*_app_* 3.618 × 10^−5^ ± 1.963 × 10^−6^ cm s^−1^).

**Figure 13:**
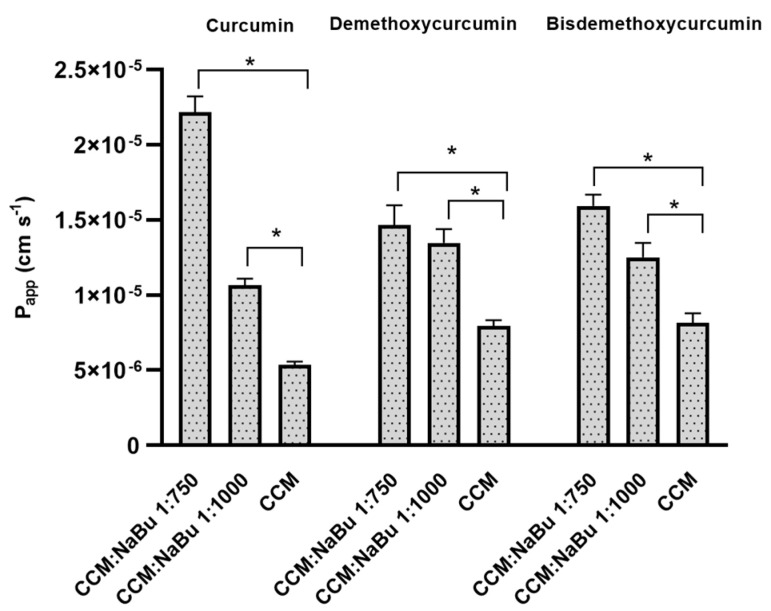
Values of apparent permeability coefficients of curcumin, demethoxycurcumin, and bisdemethoxycurcumin alone and with an addition of NaBu in molar ratio of 1:750 (20 µM CCM:15 mM NaBu) and 1:1000 (10 µM CCM:10 mM NaBu) determined for permeability through nasal cavity. Values are shown as mean ± SEM calculated from three independent experiments. (*) indicates statistically significant differences, *p* < 0.05.

According to the classification, the compounds with P*_app_* value above 1.0 × 10^−6^ cm s^−1^ are well permeable [20]. The results of our study indicate that curcumin, with the P*_app_* value of 5.337 × 10^−6^ ± 2.424 × 10^−7^ cm s^−1^ can be considered highly permeable through the nasal cavity membranes (Figure 13). However, with the addition of NaBu in a molar ratio of 1:1000, the permeability increased—P*_app_* was 1.065 × 10^−5^ ± 4.321 × 10^−7^ cm s^−1^. The greatest, more than 4-fold elevation in permeability was observed when using the combination of CCM:NaBu in a ratio 1:750 (P*_app_* 2.218 × 10^−5^ ± 1.049 × 10^−6^ cm s^−1^). Demethoxycurcumin and bisdemethoxycurcumin penetrated the nasal cavity membranes better than curcumin (P*_app_* 7.948 × 10^−6^ ± 3.693 × 10^−7^ cm s^−1^, and 8.162 × 10^−6^ ± 6.243 × 10^−7^ cm s^−1^, respectively). The use of NaBu also increased the permeability of these curcuminoids. In this context, the best results were obtained with CCM:NaBu in the ratio of 1:750, reaching P*_app_* value for demethoxycurcumin 1.466 × 10^−5^ ± 1.306 × 10^−6^ cm s^−1^, and for bisdemethoxycurcumin 1.591 × 10^−5^ ± 7.674 × 10^−7^ cm s^−1^.

## 3. Discussion

GBM is the most malignant and the most common primary brain tumor in adults, with a very poor prognosis. It is characterized by many mechanisms of treatment resistance and inevitable relapse. It shows a number of changes on the epigenetic and genetic level, leading to high invasiveness due to the infiltrative nature of growth, neoangiogenesis, and participation of stem cells resistant to cancer therapy. In addition, GBM can avoid signals leading to apoptosis, and is also characterized by hyperactivation of signaling pathways, such as Wnt/β-catenin pathway, guaranteeing continuous proliferation. Since current GBM treatments are ineffective, finding new therapeutic options are of great importance.

CCM and NaBu are natural compounds with anticancer effects and minimal toxicity. However, their molecular targets and mechanisms exerted in GBM cells are still unclear. In this study, we found that CCM and NaBu decreased the viability of GBM cells and these compounds, and when administered in concentrations higher than 1 µM of CCM and 5 mM of NaBu, acted synergistically. This is a novel finding, as to the best of our knowledge, the combination treatment of CCM and NaBu was not analyzed in GBM cells previously. Synergism between another polyphenol, quercetin, and NaBu for controlling growth of GBM cells was, however reported [21]. In another study, curcumin used as a single compound reduced GBM cell viability, inhibiting activation of the PI3K/Akt/mTOR pathway [22]. Intriguingly, curcumin did not modify the phenotype of healthy astrocytes, suggesting that this natural compound selectively targets transformed cells [22]. As far as NaBu is concerned, in a study of Nakagawa et al. [17], its physiological concentrations (0.25–4.00 mM) dose-dependently inhibited A-172 cells proliferation and invasion. Similar results were also published using a model of U251 cells [23]. Importantly, in that study, the decreased viability was also accompanied by the increased radiosensitivity of GBM cells.

To decipher what mechanisms may be responsible for the observed viability reduction of GBM cells, we performed subsequent apoptosis and cell cycle flow cytometry analyses. We found that CCM and NaBu induce apoptotic cell death; however, the degree of apoptosis was cell line-dependent. In this regard, CCM was most active in U-138 MG cells, while in T98G, it increased the percentage of only early apoptotic cells. On the other hand, NaBu acted proapoptotically in all analyzed cells, but the degree of apoptosis varied between the cell lines. Moreover, we observed significant increases in apoptotic cell number when the combination of compounds was used, and the results for combination treatment resembled that of a reference anticancer drug—topotecan. The proapoptotic effect of the tested compounds was also accompanied or could were induced by the cell cycle distribution changes. In this regard, the combination treatment of CCM and NaBu was actively arresting the cell cycle in the G2/M phase in all cell lines, while single compounds were active in two out of three analyzed cell lines, i.e., T98G and U-138 MG cells.

Next, in the attempt to unravel the molecular mechanisms underlying CCM and NaBu-mediated growth inhibition and apoptosis induction, we performed the oxidative stress analysis of the cells treated with the analyzed compounds and their combination. Here, we report that direct cytotoxic and proapoptotic effects of CCM in T98G and U-138 MG cells are mediated, at least in part, via ROS production. Our findings are in line with a study by Gersey et al. [24], who found that 25 μM curcumin significantly induced ROS in GBM stem cells. Thus, these data confirm that even though curcumin is considered a natural phenolic antioxidant, it can act pro-oxidatively in cancer cells, inducing desirable anticancer effects. Moreover, in our study also NaBu-elicited apoptosis in A-172 cells was accompanied by an elevated level of ROS. Similar results were published by Nakagawa et al. [17] and Salimi et al. [16]; however, the latter reported pro-oxidative effects of NaBu in regard to breast cancer cells. Thus, the data suggest that ROS generation may be responsible for the observed increase in the apoptotic cell number in CCM and NaBu-treated GBM cells.

The Wnt/β-catenin signaling pathway modulates the transcription of genes linked to proliferation, differentiation, and tumor progression. Targeting this pathway is suggested as a good strategy in anti-GBM treatment, as it is often hyperactivated in GBM and contributes to malignant glioma stemness, invasiveness, therapeutic resistance, and angiogenesis. Previously, we and others found that Wnt/β-catenin signaling is hyperactivated in glioma cells due to promoter methylation of its antagonists, including *SFRP1* and *RUNX3* [25]. Another frequent event in GBM cells is the epigenetic silencing of a master cell cycle regulator, *RASSF1A* [26]. Thus, we wanted to analyze if CCM and NaBu are able to reactivate *RUNX3*, *SFRP1*, and *RASSF1A* gene expression and if this change in expression is accompanied by DNA promoter demetylation. Here, we report significant upregulation of *RUNX3* (in A-172 and U-138 MG) and *SFRP1* (in all three cell lines analyzed) after the treatment with NaBu and the combination treatment of CCM and NaBu. This is an important observation since NaBu was reported to modulate the Wnt signaling in a cell type- and promoter-dependent manner [27]. For instance in SW620 colon carcinoma cells, NaBu downregulated β-catenin-dependent expression of the Tcf-TK, matrilysin, and cyclin D1 promoters [27]. Contrarily, in another colon carcinoma cell line, HCT-116, NaBu upregulated the expression of these promoters [27]. As the impact of NaBu on GBM cells in the context of Wnt signaling pathway was not analyzed so far, this novel finding is of great importance, especially since our previous study revealed that *SFRP1* methylation predicts shorter survival of glioma patients [25].

The results of our study also indicate that NaBu, and its combination treatment with CCM, upregulates the critical cell cycle regulator, *RASSF1A*, in A-172 and T98G cells, but downregulates its mRNA level in U-138 MG cells. This finding shows a cell line-dependent manner of *RASSF1A* expression regulation by CCM and NaBu, and indicates that this issue should be further analyzed.

Gene expression changes are often a result of DNA methylation changes at the promoter, enhancer, or other regulatory regions. The ability of curcumin to demethylate tumor suppressor genes was previously demonstrated [8]. On the other hand, NaBu is well known as histone deacetylase (HDAC) inhibitor, but its impact on DNA methylation was also reported [10]. Thus, we wanted to analyze if the observed gene expression changes result from demethylating properties of the studied compounds in the context of the particular, important for gliomagenesis genes. Here, we found that CCM had no demethylating or hypomethylating properties regarding *RUNX3*, *SFRP1*, and *RASSF1A* promoters in GBM cell lines analyzed in this study. In contrast, hypomethylating effects of NaBu were cell line- and gene-dependent, as it reduced the methylation level of selected genes in A-172 and T98G cell lines. Thus, we can conclude that the impact of CCM on the promoter methylation of *RUNX3*, *SFRP1*, and *RASSF1A* was inconsiderable, while of NaBu—moderate. Similar results were obtained by Parashar et al. [28], who investigated the reactivation of *PAX1* using curcumin and resveratrol in HeLa, SiHa, and Caski cell lines. They found that curcumin in HeLa and SiHa cells and resveratrol in Caski cells caused significant reactivation of *PAX1* expression; however, the reversal of promoter hypermethylation was not observed across the three cell lines. Contrarily, in a study of Shin et al. [10] NaBu induced demethylation and histone modification at the promoter region of *SFRP1/2*, restoring the SFRP expression in human gastric cancer cells. Similar to our results, in that study, NaBu did not induce complete demethylation, but generated a more sporadic pattern of demethylation.

Interestingly, in our study, even Aza, the positive control with DNMT inhibitory properties, was not found to be effective in causing complete demethylation of CpG sites under consideration, suggesting the promoter regions to be resistant towards hypomethylating effects. We discovered that Aza was able to reduce the DNA methylation levels of *RUNX3*, *SFRP1*, and *RASSF1A* of only ~25%, and did not completely demethylate any of the analyzed sequences. In this context, we can conclude that NaBu and its combination treatment with CCM had similar hypomethylating properties as Aza. Incomplete demethylation at various genomic regions after the treatment with Aza and another hypomethylating agent, 5-aza-2′-deoxycytidine (5-Aza-dc) were also reported by others [28,29]. It was shown that Aza treatment does not result in demethylation of every CpG in DNA, as some Aza-treated regions can remain methylated, even to a large extent. For instance, the presence of methylated DNA in samples treated with 5-Aza-dc was reported for genes such as *Axin1*, *CXCL12*, *DAPK1*, and others [29]. These findings can explain why curcumin (the major component of CCM used in our study), despite being frequently reported as a potent DNMT inhibitor, was not effective in demethylating the genes chosen in our experimental setting.

Since the gene expression changes observed in our study were not caused by complete promoter demethylation, we wanted to verify, if the analyzed compounds had an impact on the key epigenetic regulator—UHRF1 (ubiquitin-like with plant homeodomain and really interesting new gene finger domain 1). UHRF1 is involved in a macro-molecular protein complex called ‘Epigenetic Code Replication Machinery’ (ECREM), which is coordinating DNA methylation and histone modifications [30]. Importantly, UHRF1 is regarded as a potential target in anticancer therapy since its expression is upregulated in multiple tumors, and its downregulation was reported to lead to growth arrest and apoptosis of cancer cells [30]. Here we report that NaBu and the combination treatment of CCM and NaBu downregulated the expression of *UHRF1* in GBM cells. Contrarily, CCM upregulated its expression. Other research groups already showed that UHRF1 can be targeted by phytocompounds, including curcumin, but both downregulation and upregulation were reported, depending on the cell line analyzed and the time of exposure to the compound [28]. Regarding the complex role played by UHRF1, and the ambiguous data from different experimental setups, further studies are urgently needed to fully elucidate the impact of CCM on this epigenetic regulator and the subsequent phenomena controlled by it. Nevertheless, NaBu and the combination treatment of CCM and NaBu can be regarded as promising compounds downregulating *UHRF1* in GBM cells. We hypothesize that the anticancer effects including reduced viability, apoptosis induction, and cell cycle arrest accompanying the treatment with NaBu and the combination treatment of CCM and NaBu, can be, at least in part, related to *UHRF1* downregulation.

Finally, to verify if the two compounds can successfully be implemented as a combination treatment targeting CNS, the permeability of CCM and NaBu through the BBB and the nasal cavity was analyzed. Curcumin used as a single compound was already found to be able to cross the BBB [5], yielding therapeutic benefits within the CNS. NaBu is also able to reach CNS via the gut-brain axis. In humans, colonic butyrate can range from 10 to 20 mM [31]. Physiologically, NaBu is detectable in the human cerebrospinal fluid (CSF), typically in the range of 0–2.8 μM, and an average concentration of 17.0 pmol/mg in the human brain was reported. Thus, the physiological concentration of this microbiota-derived metabolite is far lower than that required for anticancer effects [13]. Novel delivery systems are therefore needed to expose GBM cells to the increased concentrations of both compounds. Interestingly, in this study, we found that CCM permeability through the BBB and through the nasal cavity is increased, when it is administered together with NaBu. In fact, the combination of CCM with NaBu increased the permeability of all three curcuminoids, including curcumin, demethoxycurcumin, and bisdemethoxycurcumin. This finding paves the way for novel oral, or nose-to-brain delivery systems, with better bioavailability and improved efficacy. Intranasal drug delivery systems seem especially promising, as they are not only effective in bypassing the BBB but also very convenient to use and painless. To conclude, the findings of this study give rationale for further pre-clinical and clinical analysis of the combination treatment of CCM and NaBu and suggest the great value of the implementation of CCM and NaBu-based drug candidates in anti-GBM treatment.

## 4. Materials and Methods

### 4.1. Chemicals

CCM, NaBu, Aza, topotecan, dimethylsulfoxide (DMSO), antibiotics solution (10,000 units penicillin and 10 mg streptomycin/mL), Eagle’s Minimum Essential Medium (EMEM), Dulbecco’s modified Eagle medium (DMEM), fetal bovine serum (FBS), glutamine, nonessential amino acids (NEAA), sodium pyruvate (NaP), ribonuclease A (RNase A), 3-(4,5-dimethylthiazol-2-yl)-2,5-diphenyltetrazolium bromide (MTT), trypsin, Tris, and all other compounds were purchased from Sigma–Aldrich (St. Louis, MO, USA).

CCM and Aza were dissolved with DMSO to make 10 mM stock solutions, aliquoted and frozen at −20 °C, and −80 °C, respectively. Only one freeze-thaw cycle was used regarding Aza to ensure maximal stability. For the same reason, NaBu was at all times dissolved *ex tempore* in PBS to make a 100 mM stock solution.

### 4.2. HPLC Analysis

The HPLC separation was carried out on a Phenomenex-C18 column (250 mm × 4.6 mm; 5 µm), the temperature of the column set at 303 K, using a mobile phase consisted of 1% acetic acid and acetonitrile (45:55, *v*/*v*), with the flow rate 1.0 mL/min (1). The injection volume was 20 µL, and maximum absorption was measured using Diode-Array (DAD) detector set at 421 nm. As reference standards, curcumin (purity > 99.5%), demethoxycurcumin (purity > 95.0%) and bisdemethoxycurcumin (purity > 98%) were used.

### 4.3. Cell Lines and Culture

Human GBM cell lines, A-172 and U-138 MG were obtained from American Type Culture Collection (ATCC), whereas human GBM T98G cell line was bought from the European Collection of Authenticated Cell Cultures (ECACC). The cells were maintained in the recommended media: ATCC-formulated Dulbecco’s modified Eagle’s medium (DMEM) (Merck, Darmstadt, Germany) in case of A-172 cell line, and ATCC-formulated Eagle’s Minimum Essential Medium (EMEM) (Merck, Germany), in case of T98G and U-138 MG. The media were supplemented with FBS (Biowest, Nuaillé, France) to a final concentration of 10%, as well as antibiotics (penicillin and streptomycin) (Merck, Darmstadt, Germany) to the final concentrations of 1%. For the experiments, the amount of FBS was reduced to 5%. Additionally, the medium for T98G was supplemented with 2 mM glutamine, 1% non-essential amino acids, and 1% sodium pyruvate (all purchased from Merck, Germany). The cells were propagated at 37 °C and 5% CO_2_ in a humidified incubator (Memmert, Schwabach, Germany).

### 4.4. Cytotoxic Activity

The cytotoxic activity of CCM, NaBu, and their combinations, as well as Aza, a demethylating agent used as a control for methylation analysis, was performed using the MTT assay. Briefly, cells were cultured, harvested, and plated in 96-well plate at a seeding density of 10,000 cells/well. After 24 h of preincubation, increased concentrations of CCM (0.5–20 µM), NaBu (2.5–15 mM), CCM and NaBu (0.5 µM + 2.5 mM − 20 µM + 15 mM of CCM and NaBu, respectively) or Aza (0.25–50 µM) were added, and cells were grown for 48 h at 37 °C. Appropriate controls with DMSO (for CCM, the combination treatment of CCM and NaBu, and Aza) and PBS (for NaBu) were performed. Afterwards, the cells were washed with 200 µL of PBS, followed by incubation with 3-(4,5-dimethylthiazolyl-2)-2,5-diphenyltetrazolium bromide (MTT) solution (Merck, Darmstadt, Germany) in 10% FBS medium (0.5 mg/mL) for 3 h. Finally, the formazan crystals were dissolved in acidic isopropanol, and the absorbance was measured at λ = 570 nm and λ = 690 nm on the microplate reader (Tecan Infinite M200, Grödig, Austria). All the experiments were repeated three times with four measurements per assay.

### 4.5. The Combinatorial Effects of the Compounds on Cytotoxic Activity (Combination Index)

The combinatorial effect of the compounds on cell viability was determined by the evaluation of the Combination Index (CI) using the CompuSyn software (www.combosyn.com, accessed on 18 August 2021) [32]. The synergistic action of the chemicals in combinations was identified when CI ˂ 1.

### 4.6. Apoptosis Analysis

The flow cytometry apoptosis analysis using the Muse^®^ Annexin V & Dead Cell Kit (Merck, Darmstadt, Germany) was performed following the protocol provided by the manufacturer. The principle of this method relies on Annexin V binding to phosphatidylserine—the marker of early apoptosis, while 7-Aminoactinomycin binding to DNA in late apoptotic/dead cells. Briefly, 100,000 cells of A-172 and T98G cell lines, and 150,000 cells of U-138 MG cell line were seeded on 6-well plates and incubated for 24 h. Afterward, the analyzed compounds were added in concentrations based on MTT results, and the cells were further incubated for 48 h. Cells treated with the vehicle (DMSO or PBS) and 100 nM topotecan were used as negative and positive controls, respectively. The subsequent analysis was performed on Muse™ Cell Analyzer (Merck, Darmstadt, Germany).

### 4.7. Cell Cycle Distribution Analysis

The cell cycle distribution analysis using the Muse™ Cell Cycle Kit and Muse™ Cell Analyzer (Merck, Darmstadt, Germany) was used to check the number of cells in each phase of the cell cycle. The assay utilizes propidium iodide-based staining of DNA content to discriminate and measure the percentage of cells in each cell cycle phase (G0/G1, S, and G2/M). The analysis was performed according to the protocol provided by the manufacturer and was previously described [33]. Briefly, after 48 h incubation with the analyzed compounds, the cells were trypsinized, washed with PBS buffer, fixed in ice-cold 70% ethanol, and stored until staining at −20 °C. Before the analysis, the fixed cells were washed with PBS buffer, stained, and subjected to 0.5 h incubation at room temperature in the darkness. Cells treated with the vehicle (DMSO or PBS) and 100 nM topotecan were used as negative and positive controls, respectively. The subsequent analysis was performed on Muse™ Cell Analyzer (Merck, Germany).

### 4.8. Oxidative Stress

The intracellular detection of superoxide radicals was performed using The Muse^®^ Oxidative Stress Kit (Merck, Darmstadt, Germany), according to the manufacturer’s recommendations. The principle of the method is based on dihydroethidium (DHE), which is cell permeable and upon reaction with superoxide anions undergoes oxidation to form the DNA-binding fluorophore. The fluorophore intercalates with DNA which results in red fluorescence. Briefly, the cells were treated with the analyzed compounds/mixes, in the concentrations allowing >70% of cell survival. After 48 h the cells were trypsinized, washed with PBS buffer, and resuspended in Assay Buffer containing Working Solution of Muse^®^ Oxidative Stress Reagent. The cells were incubated at 37 °C for 30 min, and then run on Muse^®^ Cell Analyzer (Merck, Darmstadt, Germany). Cells treated with the vehicle (DMSO or PBS) were used as negative control.

### 4.9. cDNA Synthesis & qPCR

The total RNA was isolated using Universal RNA Purification Kit (EURx, Gdańsk, Poland) and subsequently subjected to reverse transcription using The Revert-Aid First Strand cDNA Synthesis kit (Fermentas, Burlington, Canada). Quantitative real-time PCR (qPCR) was performed using Hot FIREPol EvaGreen qPCR Mix Plus (Solis Biodyne, Tartu, Estonia) and LightCycler 96 (Roche Diagnostics GmbH, Mannheim, Germany). Primer sequences were previously published [3,28,34,35], and their synthesis was performed at the Institute of Biochemistry and Biophysics, Warsaw, Poland. The protocol started with 15 min enzyme activation at 95 °C, followed by 40 cycles of 95 °C for 15 s; appropriate annealing temperature for 20 s; 72 °C for 40 s and the final elongation at 72 °C for 5 min which was followed by melting curve analysis to confirm the generation of a single amplification product. TATA box-binding protein (*TBP*), a component of the DNA-binding protein complex TFIID, served as the endogenous RNA control, and each sample was normalized on the basis of its *TBP* content. The results were expressed as N-fold differences in gene expression relative to the *TBP* gene. Each sample was analyzed in triplicate, and the experiments were repeated twice.

### 4.10. Sodium Bisulfite Treatment & Methylation-Sensitive High-Resolution Melting Analysis (MS-HRM)

EZ DNA Methylation Kit (Zymo Research, Irvine, CA, USA) was used for sodium bisulfite treatment, i.e., deamination of unmethylated cytosines within CpG dinucleotides to uracil while leaving the 5-methylcytosines intact. Such bisulfite-converted DNA was subsequently subjected to MS-HRM analysis. All the methodological details of the MS-HRM analyses including the reaction and primer sequences can be found elsewhere [3,34], and we previously validated the MS-HRM protocols used in this study with pyrosequencing, which is considered the gold standard technique for DNA methylation investigations. Briefly, the standard solutions of completely methylated genomic DNA, i.e., CpG Methylated HeLa Genomic DNA (New England Biolabs, Ipswich, MA, USA) and completely unmethylated human genomic DNA, i.e., CpGenome Universal Unmethylated DNA Set (Merck, Darmstadt, Germany) were used to generate melting curves of known methylation level (100%, 75%, 50%, 25%, and 0% methylated DNA). To semiquantitatively estimate the sample methylation levels, their sample melting profiles were compared to those of standards. Samples were run in duplicate in each experiment, and each experiment was repeated twice. The protocol involved 15 min of preincubation at 95 °C and 40 cycles of three-step amplification (15 s/95 °C, 20 s/Ta, 20 s/72 °C), and obtained amplicons were melted in a temperature gradient to max 95 °C. The obtained melting curves were normalized automatically by the calculation of the “line of the best fit” in between two normalization regions before and after the significant fluorescence decrease. The methylation level of each sample was assessed by comparison of the PCR product normalized melting curve/peak with the normalized melting curves/peaks of the standard solutions.

### 4.11. PAMPA Model

The impact of NaBu on the permeability of CCM (curcumin, and two other curcuminoids—demethoxycurcumin and bisdemethoxycurcumin) through the BBB and the wall of nasal cavity was investigated using the Parallel Artificial Membrane Permeability Assay model (PAMPA). The model defines the permeability basing on the passive diffusion across the BBB and nasal mucosa. The PAMPA set consists of donor and acceptor plates, separated by a 120 μm-thick microfilter disc coated with a 20% (*w/v*) dodecane solution of a lecithin mixture (Pion Inc., Billerica, MA, USA). Prisma buffers (Prisma HT, Pion Inc.) of pH 7.4 and pH 6.0 were used in the BBB permeability and nasal cavity permeability tests, respectively. The samples containing curcuminoids were studied alone and with NaBu in molar ratio CCM:NaBu 1:750; and 1:1000. The samples were distributed to donor wells with appropriate Prisma buffer, and the acceptor wells for nasal and BBB permeability were filled with Acceptor Sink Buffer, and Brain Skin Buffer (Pion Inc., Billerica, MA, USA), respectively. Then, the plates were combined to form a sandwich. The nasal PAMPA plate was incubated for 1 h, while PAMPA BBB was incubated for 4 h in a humidity-saturated atmosphere at the temperature set at 37 °C. After incubation, the plates were separated to investigate curcuminoids concentrations in donor and acceptor chambers using the HPLC-DAD method, as described above (Section 4.1). The apparent permeability coefficient (P*_app_*) was calculated due to the equation:

$$P_{app}=\frac{-ln\left(1-\frac{C_{A}}{C_{equilibrium}}\right)}{S\times \left(\frac{1}{V_{D}}+\frac{1}{V_{A}}\right)\times t}$$

where *V_D_*—donor volume, *V_A_*—acceptor volume, *C_equilibrium_*—equilibrium concentration $C_{equilibrium}=\frac{C_{D}\times V_{D}+C_{A}\times V_{A}}{V_{D}+V_{A}}$, *S*—membrane area, *t*—incubation time (in seconds).

### 4.12. Statistical Analysis

All data are present as the mean and standard error of the mean (SEM). Differences among experimental groups were determined by the unpaired *t*-test with two-tailed distribution. In all comparisons, statistical significance was set at *p* < 0.05.

## Data Availability

Data supporting reported results can be found at the database of Poznan University of Medical Sciences.
